# Life Cycle Assessment of Completely Recyclable Concrete

**DOI:** 10.3390/ma7086010

**Published:** 2014-08-21

**Authors:** Mieke De Schepper, Philip Van den Heede, Isabel Van Driessche, Nele De Belie

**Affiliations:** 1 Magnel Laboratory for Concrete Research, Department of Structural Engineering, Ghent University Technologiepark-Zwijnaarde 904, B-9052 Gent, Belgium; E-Mails: midschep.deschepper@ugent.be (M.S.); philip.vandenheede@UGent.be (P.H.); 2 SCRiPTS, Department of Inorganic and Physical Chemistry, Ghent University, Krijgslaan 281–S3, B-9000 Gent, Belgium; E-Mail: isabel.vandriessche@ugent.be

**Keywords:** Life Cycle Assessment, Completely Recyclable Concrete, Construction & Demolition Waste, recycling

## Abstract

Since the construction sector uses 50% of the Earth’s raw materials and produces 50% of its waste, the development of more durable and sustainable building materials is crucial. Today, Construction and Demolition Waste (CDW) is mainly used in low level applications, namely as unbound material for foundations, e.g., in road construction. Mineral demolition waste can be recycled as crushed aggregates for concrete, but these reduce the compressive strength and affect the workability due to higher values of water absorption. To advance the use of concrete rubble, Completely Recyclable Concrete (CRC) is designed for reincarnation within the cement production, following the Cradle-to-Cradle (C2C) principle. By the design, CRC becomes a resource for cement production because the chemical composition of CRC will be similar to that of cement raw materials. If CRC is used on a regular basis, a closed concrete-cement-concrete material cycle will arise, which is completely different from the current life cycle of traditional concrete. Within the research towards this CRC it is important to quantify the benefit for the environment and Life Cycle Assessment (LCA) needs to be performed, of which the results are presented in a this paper. It was observed that CRC could significantly reduce the global warming potential of concrete.

## 1. Introduction

It is known that the concrete industry has a considerable impact on the environment. Every year, 10 billion tons of concrete are produced [1]. To produce this concrete, a large amount of natural resources is consumed. For example, 42% of the aggregates annually produced, is used for concrete production [2]. In addition, the cement industry is a big user of the Earth’s raw materials. For the production of 1 kg of cement, 1.6 kg of raw material are needed [3]. Another impact of the concrete industry on the environment is related to the production of waste. Yearly the European construction sector produces 850 million tons of waste, which represents 31% of the total waste generation [4]; 40%–67% of the Construction and Demolition Waste is concrete [5,6]. Besides this, the cement industry is known for its high CO_2_ emissions: worldwide 1.6 billion tons of CO_2_ each year, which is around 8% of the total CO_2_ emissions from human activities [6].

For the above mentioned reasons researchers are making efforts to study recycling opportunities in concrete and cement production. After all, recycling has three benefits: it reduces the demand for new resources, it cuts down on production energy costs, and it recycles waste which would otherwise be landfilled [7]. For instance, over the years alternative binders like blast furnace slag, fly ash, or silica fume, which are industrial by-products, have become valuable materials for concrete production. Besides the benefits regarding the waste production and the use of raw materials, also the CO_2_ emissions can be lowered significantly as less cement is needed for concrete production. To tackle the problem regarding the waste production of the concrete industry, a great deal of research has been done regarding the use of concrete rubble as aggregate. Despite these efforts, demolished concrete is today still mostly used as aggregates in granular base or sub-base applications, for embankment constructions and in earth construction works [8]. The main problem is that the mortar and cement paste attached to the old stone particles, increase the water absorption and reduce the abrasion resistance (Los Angeles test) [8].

As an alternative of using concrete rubble as recycled aggregates, a project was started to study the possibilities of Completely Recyclable Concrete (CRC). This concrete is designed to be recycled within the cement production without need for adjustments, *i.e.*, it is possible to produce cement with CRC being the single ingredient. To serve this purpose, the chemical composition of CRC needs to be the same as the one of a traditional cement raw meal. Portland Clinker consists for about two thirds of calcium oxide (CaO), which makes it necessary to incorporate limestone aggregates into CRC. The second most important oxide is silicon oxide (SiO_2_) which is found in sand and fly ash. The other components, aluminium (Al_2_O_3_) and iron (Fe_2_O_3_) oxide, can be provided by porphyry aggregates, copper slag or calcium aluminate cement. Further reading on how this CRC is designed can be found in De Schepper *et al.* [9]. This CRC is designed to reduce the impact of concrete on the environment, and obviously it is necessary to prove the benefit for the environment through life cycle assessment. The results of a life cycle assessment on two CRCs are presented within this paper.

## 2. Goal and Scope Definition

### 2.1. Goal

The aim of this study is to quantify the impact of two CRC mixtures (CRC1 and CRC2) on the environment. Their impact will be evaluated in comparison with the reference concrete mixtures T(0.50) or T(0.45). The compositions of the studied concretes are given in Table 1.

The idea for CRC came from the finiteness of natural resources and wants to optimize concrete recycling opportunities by designing it as an ultimate raw meal for cement production. It is, thus, hoped that the implementation of CRC will reduce the demand for primary raw materials. Furthermore it is hoped to decrease the global warming potential due to the use of by-products such as fly ash and blast furnace slag, reducing the clinker content of the concrete.

**Table 1:** Overview of the compositions of the studied concrete types (kg/m^3^).

Material	CRC1	CRC2	T(0.50)	T(0.45)
Siliceous sand 0/4	-	-	714	715
Limestone sand 0/4	764	844	-	-
Copper slag 0/4	44	33	-	-
Gravel 2/8	-	-	515	515
Limestone aggregate 2/6	443	361	-	-
Gravel 8/16	-	-	671	671
Limestone aggregate 6/20	532	410	-	-
CEM I 52.5 N	300	-	320	340
CEM III/A 42.5 N LA	-	325	-	-
Fly ash	100	85	-	-
Limestone filler	53	177	-	-
Water	153	154	160	153
SP ^a^	10	17	-	5

^a^ SP = superplasticizer, Glenium 51 concentration 35% (BASF, Ham, Belgium) (mL/kg cement).

### 2.2. Scope

**Figure 1:**
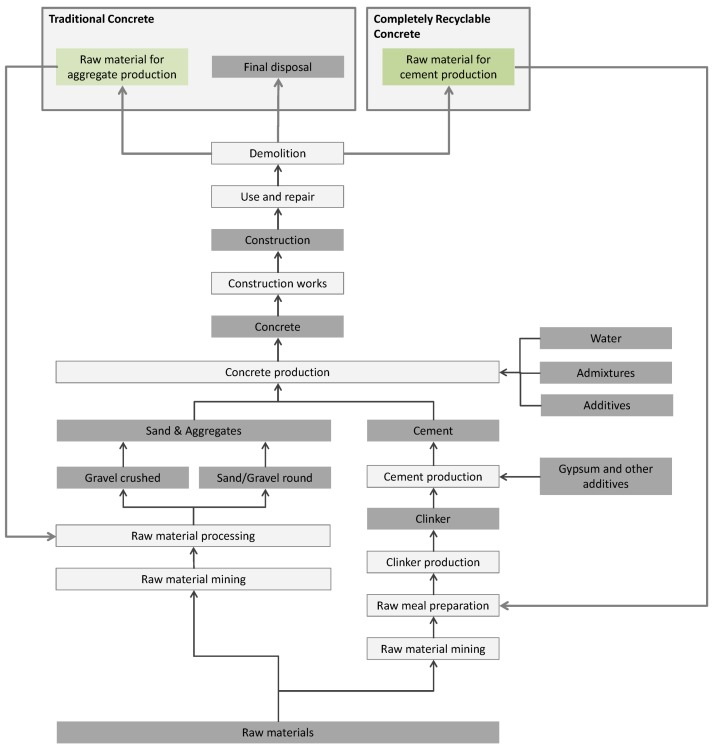
Life cycle of concrete.

To perform a proper study regarding the environmental impact of products, it is essential to make an unambiguous definition of its scope. In this study the whole life cycle of concrete will be considered (see Figure 1), starting with the exploitation of the raw materials and ending with the recycling or disposal of the demolished concrete, taking into account the (positive) impact of avoiding the use of natural raw materials. The environmental impact of the application field and repair of the structure are considered into the functional unit which takes into account the strength and durability performance of the concrete mixtures. However, the environmental impact related to the use phase, e.g., the effect on the energy consumption for heating of the building, is not considered.

### 2.3. Functional Unit

The functional unit is seen as the reference unit of the product system for which the environmental impact will be calculated. Within this study it was decided not to compare 1 m^3^ of traditional concrete with 1 m^3^ of CRC, but to compare the total amount of concrete necessary to deliver 1 MPa of strength and one year of service life. This definition of the functional unit is analogous to the definition of the binder intensity proposed by Damineli *et al.* [10], for which an adaption was suggested by Van den Heede and De Belie [11] to obtain a unit of functional performance on two levels, namely strength and durability/service life.

#### 2.3.1. Strength Performance

Using concrete with a higher strength could reduce the amount of concrete needed to obtain the same performance of a certain construction. Nonetheless, it should be noted that the amount of concrete that can be saved is highly dependent on the type of the structural element. By Habert and Roussel [12] it was found that for a concrete structure, a vertical element is the most environment friendly. The compressive strengths of CRC1, CRC2, T(0.50), and T(0.45) can be found in Table 2.

**Table 2:** Overview of the calculated Functional Units

Concrete mixture	Compressive strength (MPa)	*t*_service_ (years)	Functional unit (m^3^ concrete per MPa per year service life)
CRC1 with *n* = 0.5 after 2 months curing	65.1	81	1.90 × 10^−4^
CRC1 with *n* = 0.5 after 4 months curing	65.1	63	2.44 × 10^−4^
CRC1 from all other calculations	65.1	100	1.53 × 10^−4^
CRC2	84.2	100	1.19 × 10^−4^
T(0.50)	57.8	100	1.73 × 10^−4^
T(0.45)	69.3	100	1.44 × 10^−4^

#### 2.3.2. Durability/Service Life

Both CRC and traditional concrete mixtures were designed according to NBN EN 206-1 [13] and NBN B15-001 [14]. The reference mixtures T(0.50) and T(0.45) were produced as reference for the environmental classes XC4 (carbonation induced corrosion in a cyclic wet and dry environment) and XS3 (chloride induced corrosion from sea water in tidal, splash, and spray zones) respectively. For both CRC mixtures, the k-value concept is applicable, whereby their performance is expected to be comparable to T(0.45). Their application is thus allowed in both environmental classes XC4 and XS3. For this reason, with an appropriate concrete cover and field of application (XC4 and/or XS3 environment), a service life of 100 years should be guaranteed for all four concrete mixtures.

Nonetheless durability tests were performed to verify the potential service life for concrete exposed to carbonation or chlorides, whereby rebar corrosion can be initiated. The results of this durability assessment can be found in De Schepper [15]. Based on these results, service times were predicted in De Schepper [15] (see Table 2). In case of carbonation-induced corrosion, it was concluded that depending on the exponent used in the carbonation model (*x* = *k*·*t^n^*; with *x* the carbonation depth (mm), *k* the carbonation coefficient, *t* the exposure time [weeks] and *n* = 0.4 or 0.5) the service time for CRC1 varied from 63 to more than 100 years. For CRC2, T(0.50) and T(0.45) service times of at least 100 years were obtained for all calculations. Regarding chloride induced corrosion, the service life of 100 years was found valid for all four mixtures.

#### 2.3.3. Calculation of the Functional Unit

With the obtained service times the functional units were calculated. Within the calculations the service time was given a maximum value of 100 years since this was the designed minimum life span. The results are presented in Table 2.

## 3. Life Cycle Inventory

Most of the data used in this study were taken from the ecoinvent 2.0 database [16], which was built by the Swiss Centre for Life Cycle Inventories and is commonly used in combination with the LCA software used (SimaPro, Amersfoort, The Netherlands, see Section 4). Since adaptations were necessary to take into account, e.g., the recycling of CRC within the clinker manufacturing process, a brief overview of the changes made is given hereafter. The actual data used can be found in De Schepper [15].

### 3.1. Cement Production Process

Within the ecoinvent 2.0 database [16], the cement production process is divided into two sub processes, namely the clinker production (burning of the clinker raw materials) and the actual cement production (milling of the cement raw materials). The data for both Ordinary Portland Cement and Blast Furnace Slag Cement were taken from the ecoinvent 2.0 database [16]. The clinker production process was split into the raw material delivery and the actual clinker production to enable the recycling possibilities of CRC within the clinker production process (see Section 3.4). Within the raw material delivery process, not only the raw material extraction and transport were considered, but also the CO_2_ emissions related to the chemical decarbonation of limestone.

### 3.2. Concrete Production Process

The process “Concrete, normal, at plant/CH U” from the ecoinvent 2.0 database [16] was adapted to the concrete compositions of CRC1, CRC2, T(0.45) and T(0.50). Data regarding the superplasticizer were obtained from the environmental declaration published by the European Federation of Concrete Admixture Associations (EFCA) [17].

The life cycle inventory considered for the fly ash is based on [11]. Fly ash is a by-product from coal fired furnaces (e.g., for the production of electricity). The environmental impact of the coal fired furnaces should thus be partitioned between the electricity produced on the one hand and the fly ash obtained on the other hand. The partitioning of the impact can occur through allocation by mass or by economic value. In case of fly ash, a mass or economic allocation coefficient of respectively 12.4% or 1% should be applied. In this study, the economic allocation was chosen over the mass allocation since this principle takes into account the (economic) value of the products. Additionally, the enormous environmental impacts imposed when using mass allocation for the by-products might discourage the concrete industry to continue applying them as cement replacement.

The replacement of 20%–30% of natural concrete aggregates by recycled concrete aggregates will have no significant impact on the durability performance of concrete [18,19]. For this reason, the aggregate composition of T(0.45) and T(0.50) is divided into a first part that can be replaced by recycled aggregates (25% of the total aggregate content), and a second part that cannot be replaced, to guarantee the concrete durability (75% of the total aggregate content). The aggregate production process for the recycled concrete aggregates is also divided into the actual aggregate production process and the raw material delivery.

### 3.3. Construction and Use Phase

The concrete produced at the concrete mixing plant is subsequently transported to the construction site. According to De Herde and Evrard [20], the average distance from the concrete mixing plant to a construction site is 50 km and thus an additional input of 120 tkm “Transport, lorry 20–28 t, fleet average/CH U” from the ecoinvent 2.0 database [16] for 1 m^3^ concrete was considered. The energy needed for placing and compaction of the concrete was neglected according to De Herde and Evrard [20]. It should, however, be mentioned that the energy needed for compaction of concrete can be avoided when using self-compacting concrete (SCC). For a producer of concrete products this can result in significant savings on a yearly basis. De Schutter *et al.* [21] estimated that a concrete pipe factory can save annually about 1 GWh of energy when the shift is made from traditional concrete to SCC. The impact related to repair and maintenance activities are considered in the functional unit (see Section 2.3).

The environmental impact of concrete within the use phase (occupancy and operation (e.g., heating/cooling/lighting)) is highly depending on the type and application of the built concrete structure or component. These issues are of particular interest in case different construction materials are studied, in example for a specific case study. Since this paper focuses on the material of concrete itself, the environmental impact of the use phase was not considered since a comparable impact is expected for the different concrete types.

### 3.4. Demolition and End-of-Life Scenario

The data for demolition of the construction and sorting of the waste was taken from the ecoinvent 2.0 database [16], namely “Disposal, building, concrete gravel, to sorting plant/CH U”. Necessary adaptations were related to transport and the products that are avoided due to recycling opportunities. For both CRC and traditional concrete, a different waste scenario was defined.

#### 3.4.1. CRC Recycling Scenario

For CRC, all concrete waste is recycled within the cement production process. Since the geographical spread of concrete mixing plants and sorting plants was found comparable, the average distance between the construction site and a sorting plant was also assumed to be 50 km. The average minimum distance from a Flemish sorting plant to a cement plant was calculated to be 90 km.

Due to the presence of non-carbonate CaO, mainly from the cement, a lower raw material CO_2_ emission is expected within the regeneration process of cement from CRC instead of a traditional cement raw meal. Thermogravimetric analysis of CRC raw meal showed a total mass loss of about 35 wt%. The weight loss of 30 wt% between 600 and 900 °C was considered to be CO_2_. The raw material CO_2_ emissions from CRC are, thus, expected to be 0.46 g/g clinker, which is indeed about 15% lower than 0.54 g/g clinker in case of the traditional clinker production process. In practice, part of the CRC will be carbonated, and higher CO_2_ emissions can be expected. However, since this CO_2_ was captured from the atmosphere during the life cycle of concrete, this additional CO_2_ release was considered neutral in the life cycle assessment. Finally about 1.54 kg CRC will be needed for the production of 1 kg clinker, since the total mass loss measured by thermogravimetric analysis was about 35 wt%.

#### 3.4.2. Traditional Recycling Scenario

The waste scenario of traditional concrete was analysed according to two alternative routes, namely recycling as concrete aggregate or final disposal, e.g., as (sub)base material, for embankment constructions and in earth construction works. For the final disposal of concrete waste (including transport), the data were taken from the ecoinvent 2.0 database [16], namely “Disposal, building, concrete gravel, to final disposal/CH U”. Regarding the recycling of traditional concrete as aggregate raw material, the “Disposal, building, concrete gravel, to sorting plant/CH U” process in the ecoinvent 2.0 database [16] needs an additional input. Compared to round gravel, recycled concrete aggregates require a crushing process. The extra data needed were obtained from the difference between the “Gravel, crushed, at mine/CH U” process and the “Gravel, round, at mine/CH U” process from the ecoinvent 2.0 database [16]. Again, a transport distance of 50 km between the construction site and sorting plant was taken into account.

As already mentioned, concrete is able to capture CO_2_ from the atmosphere, which can be seen as a benefit when performing a Life Cycle Assessment. In case of a built concrete structure, the ability for capturing CO_2_ is negligible, however in case of demolished concrete, the impact is more significant. Collins [22] estimates that the CO_2_ emissions can be overestimated by 13%–48% depending on the type of concrete and application of the recycled concrete aggregate (RCA) during the secondary life. Since this impact is not easily quantified and is highly depending the type of concrete and application of the RCA, this CO_2_ capture from the atmosphere was not considered within the traditional recycling scenario.

## 4. Life Cycle Impact Assessment

For the impact assessment the LCA software SimaPro was used. This software is developed by PRé Consultants (Product Ecology Consultants) in the Netherlands and it contains the complete ecoinvent 2.0 database [16]. The assessment was performed according to the CML (Centre of Environmental Science of Leiden University) 2002 problem oriented impact method. For each impact category, a category indicator can be calculated based on the applicable characterisation model and the characterisation factors derived from the underlying model. An overview of the considered baseline impact categories and their characterisation factor is given in Table 3.

**Table 3:** Overview of the considered impact categories within the life cycle assessment according to the CML 2002 impact method

Impact category	Characterisation factor (Unit)
Abiotic depletion	*Abiotic depletion potential (ADP)* (kg Sb eq)
	Depletion of natural non-living resources (minerals and fossil fuels)
Acidification	*Acidification potential (AP)* (kg SO_2_ eq)
	Covers all impacts on soil, water, organisms, ecosystems & materials by acidifying pollutants (e.g., SO_2_, NO*_x_*, NH*_x_*)
Eutrophication	*Euthrophication (EP)* (kg 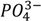 eq)
	Covers all impacts of excessively high environmental levels of macronutrients (N, P) causing a shift in species composition and an elevated biomass production in aquatic and terrestrial ecosystems
Climate change	Global warming potential (GWP 100^*^) (kg CO_2_ eq)
	Deals with all GHGs that may cause the earth’s temperature to rise and have an adverse effect on the ecosystem and human health and material welfare
Stratospheric ozone depletion	*Ozone depletion potential (ODP)* (kg CFC-11 eq)
	The ozone depletion produced by e.g., CFCs
Human toxicity	*Human toxicity potential (HTP)* (kg 1.4-DB eq)
	Covers the impact on human health of all toxic substances emitted to air, water and soil
Ecotoxicity	*Fresh water aquatic, marine aquatic and terrestrial ecotoxicity (FAETP, MAETP and TETP)* (kg 1.4-DB eq)
	Covers impacts on aquatic & terrestrial ecotoxicity of all toxic substances emitted to air, water and soil
Photo-oxidant formation	*Photochemical ozone creation potential (POCP)* (kg C_2_H_4_)
	Indicates the potential capacity of a volatile organic substance to produce ozone

^*^ Global warming potential calculated over a time interval of 100 years.

### 4.1. Cement Production Process

**Figure 2:**
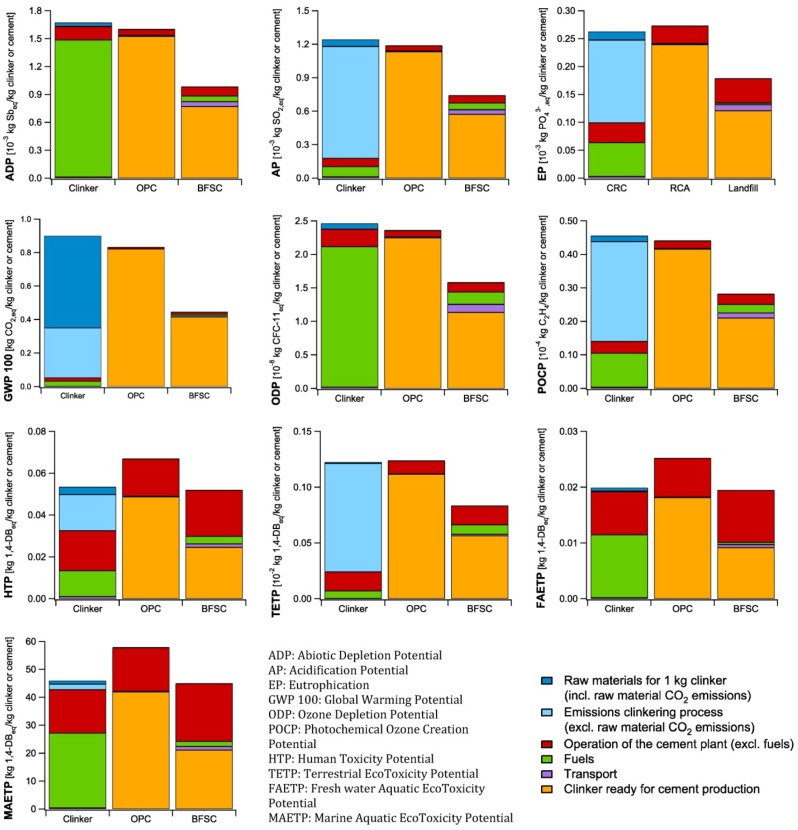
Life Cycle Impact Assessment of 1 kg clinker or cement

The results of the life cycle impact assessment of the cement production process for the different impact categories are presented in Figure 2. Three processes are visualized: the clinkering process and the manufacturing of Ordinary Portland Cement (OPC) and Blast Furnace Slag Cement (BFSC). The environmental impact of recycling CRC in the clinker production process is considered in the end-of-life scenario for CRC. The different categories related to the environmental impact of the clinker used for the manufacturing of OPC and BFSC are grouped and presented as “clinker in cement production” in Figure 2.

Taking a first look at the results, it is seen how the environmental impact of the cement production process can be reduced by incorporating supplementary cementitious materials, such as Blast Furnace Slag. Depending on the considered impact category, the environmental impact of the cement production can be reduced with 22%–47% by making use of BFSC instead of OPC. When replacing part of the clinker by BFS, only some heat (from fuels) and additional energy for grinding (in operation of the cement plant) is required for its pre-treatment. The environmental impact of this treatment is of course significantly lower compared to the whole clinkering process, explaining the reduced environmental impact of BFSC.

The cement production process is known for its high CO_2_ emissions, related primarily to the clinkering process. Looking at Figure 2, it is seen that 61% of the CO_2_ emissions for clinker production are related to the decarbonation process of the raw materials (“raw materials for 1 kg clinker (including raw material CO_2_ emissions)”). Additionally 36% is related to the burning of fuels; 33% from alternative fuels of which the CO_2_ contribution is added to the clinkering process (“emissions clinkering process (excluding raw material CO_2_ emissions)” in Figure 2) and 3% from fossil fuels (“Fuels” in Figure 2). By using BFSC instead of OPC, the Global Warming Potential of cement can be reduced by 47%.

For the other impact categories, it is seen that the environmental impact is dominated by the use of fuels and the air emissions related to the clinkering process. Furthermore the required electricity (mainly for milling of the raw materials and cement) and the consumables (e.g. ammonia, lubricating oil, refractory bricks, …) necessary for the operation of the cement plant contribute significantly to the human toxicity and aquatic ecotoxicity, and, to a smaller extent, also to the terrestrial ecotoxicity and eutrophication of the environment.

### 4.2. Concrete Production Process

**Figure 3:**
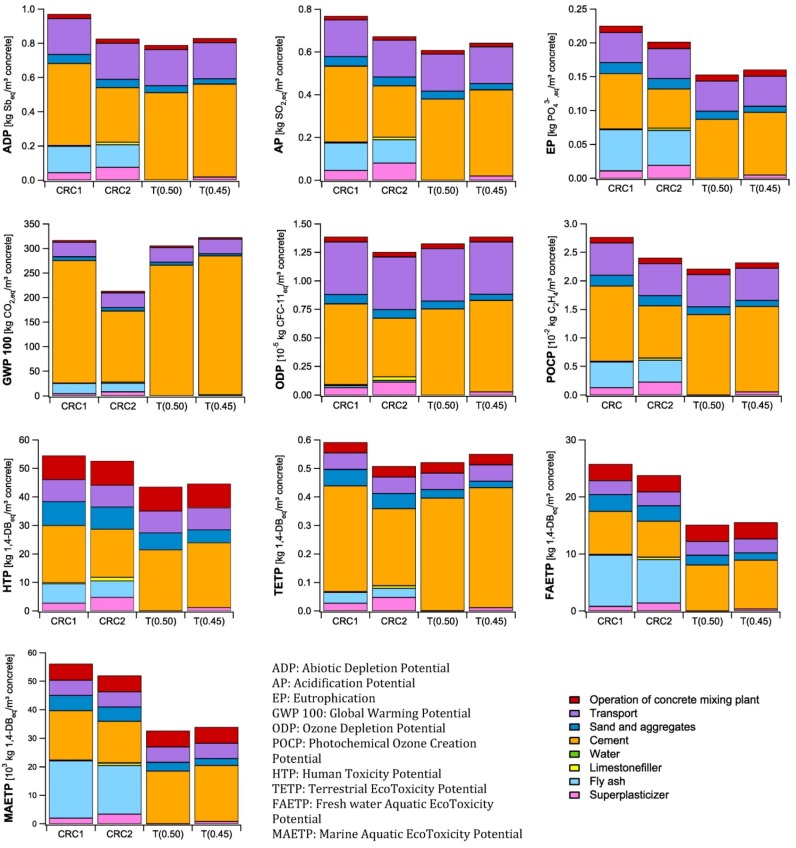
Life Cycle impact Assessment of 1 m^3^ concrete.

In Figure 3, the results of the life cycle impact assessment of the different concrete mixtures for 1 m^3^ at a construction site are shown. The Functional Unit is thus not considered yet, but the transport from the concrete plant to the construction site is incorporated into the calculations.

It is seen that depending on the considered impact category, both CRC and traditional concrete mixtures can have the lowest environmental impact. The main advantage of using CRC2 is related to the global warming potential, which is 30%–34% lower compared to the traditional concrete mixtures. The good performance of CRC2 regarding the global warming potential is due to its reduced clinker content by using both fly ash and Blast Furnace Slag Cement. Considering global warming, the environmental impact of CRC1 and the traditional concrete are comparable.

Regarding the Ozone layer depletion, CRC2 has a slightly better performance compared to the reference concrete mixtures, with a reduction of 6%–10%. Again, the result for CRC1 is comparable with the traditional concrete mixtures. While the global warming potential is strongly related to the cement content of the concrete, the ozone layer depletion is not only related to the cement content, but also to the required transport of the materials.

The environmental impact of the concrete mixtures regarding the other impact categories is mainly related to their cement and fly ash content and the required transportation. To a smaller extent also the sand and aggregates, the superplasticizer, if needed, and the operation of the concrete mixing plant contribute to these impact categories. In case of abiotic depletion, acidification, photo-oxidant formation and the terrestrial ecotoxicity, the environmental impact of CRC2, T(0.45) and T(0.50) are comparable, while the impact of CRC1 is 8%–26% higher. The environmental impact considering eutrophication, human toxicity and aquatic ecotoxicity of both CRC mixtures is worse compared to the traditional concrete mixtures. The impacts are 25%–47%, 18%–25%, and 53%–72% higher, respectively.

### 4.3. Demolition and End-of-Life Scenario

**Figure 4:**
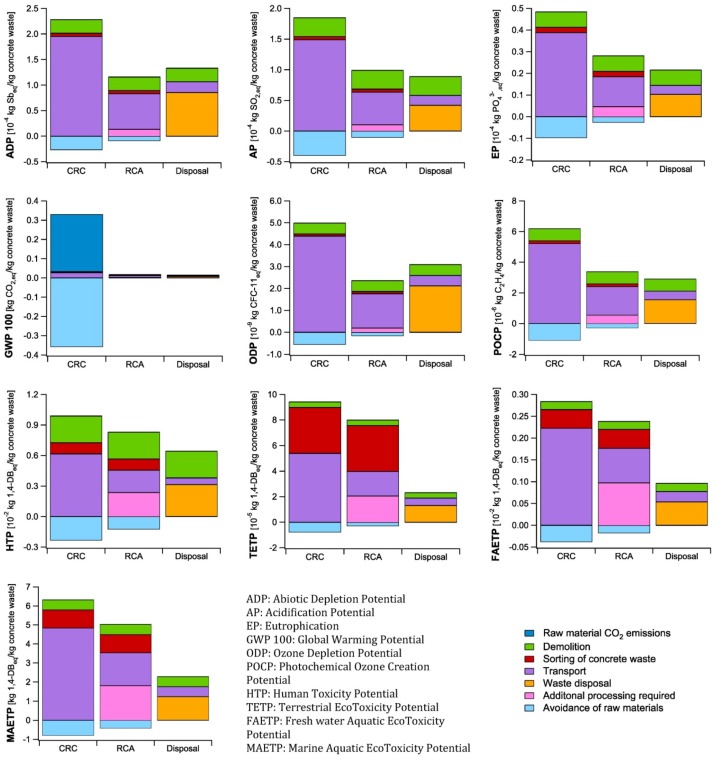
Life Cycle Impact Assessment of the different waste scenarios: CRC = Completely Recyclable Concrete; RCA = Recycling of traditional concrete as Recycled Concrete Aggregates; disposal = disposal of traditional concrete waste

The results of the impact assessment of the end-of-life scenarios for both CRC and traditional concrete mixtures are presented in Figure 4. The negative impacts observed are related to the avoidance of natural raw materials by the recycling of concrete. For the final disposal of concrete, e.g., as (sub)base material, for embankment constructions and in earth construction works, no benefit was considered since it is more a useful disposal than real recycling of the concrete waste. For the recycling of CRC as raw material for the clinker production and traditional concrete as raw material for the aggregate production, their environmental impact is lowered by 8%–24% and 4%–15% respectively, depending on the considered impact category (“Avoidance of raw materials” in Figure 4).

The results regarding global warming definitely stand out for the CRC recycling opportunity. Although the CO_2,eq_ emissions are indeed several times higher when CRC is recycled in the clinkering process, the release of a significant amount of CO_2_ is also avoided. Indeed, for each 1 kg CO_2,eq_ released upon the recycling of CRC in a clinker kiln, the emission of 1.08 CO_2,eq_ related to the burning of a traditional cement raw meal is avoided. The latter will reduce the impact on global warming of the CRC concrete mixtures significantly.

Nonetheless, for the other impact categories, the recycling of the concrete waste seems not always better compared to its final disposal. The main contributor to the environmental impact of the recycling of both CRC and traditional concrete is the transport of the materials. In total, 140 and 50 km of road transport was considered for the recycling of CRC and traditional concrete, respectively, while the ecoinvent 2.0 data used for the final disposal of concrete takes into account a transport distance of only 15 km. Indeed, concrete rubble is often disposed in the neighbourhood as (sub)base material, for embankment constructions and in earth construction works. Depending on the considered impact category, the transport contributes for 57%–88% and 24%–66% to the environmental impact of the recycling of CRC and traditional concrete respectively, while it only amounts to 10%–25% in case of the mere final disposal of the concrete waste. It will, thus, be beneficial for both recycling of CRC and traditional concrete to reduce the environmental impact of the transport by minimizing road transport and maximizing rail and barge transport. As can be expected, the main impact of the final disposal of concrete is related to its disposal to an inert material landfill (47%–69%).

The impact of the demolition process is identical for all end-of-life scenarios and amounts to 8%–27%, 5%–32%, and 16%–41% in case of CRC recycling, recycling of traditional concrete as aggregates, or final disposal of concrete waste, respectively. The additional processing needed to sort the waste materials for both recycling options is rather limited, except for the impact categories eutrophication, human toxicity, aquatic ecotoxicity and terrestrial ecotoxicity, wherein contributions of 5%–38% and 9%–45% were calculated for CRC and traditional concrete recycling, respectively. The additional processing that should be considered for the aggregate production in case of the recycling of traditional concrete varies between 8% and 41%, depending on the considered impact category.

## 5. The Environmental Impact of CRC *versus* Traditional Concrete

In the previous part of this chapter it was seen that most impact categories of the concrete production process are dominated by the cement manufacturing process and the required transportation. In case of abiotic depletion, acidification, eutrophication, photo-oxidant formation, human toxicity and aquatic toxicity, the environmental impact of fly ash might not be underestimated. The environmental impact of the sand and aggregates used in concrete is limited.

The underlying cause of the huge environmental impact of the cement production is the clinkering manufacturing. Indeed, high amounts of raw materials and fuels are needed for this process, which goes along with high CO_2_ emissions. The main goal for the development of a sustainable concrete is thus minimizing its clinker content (reducing the environmental impact of 1 m^3^ concrete), while still obtaining a high performance (lowering total amount of concrete needed to deliver 1 MPa of strength and one year of service life).

Additionally, the recycling possibilities of concrete will affect its sustainability. It was seen that additional transport and processing costs are needed for the recycling of concrete, nonetheless, natural resources will be saved and waste disposal is avoided. Comparing the recycling of CRC and traditional concrete, it was observed that although the global warming potential is lower, mainly the longer transport distances in case of CRC increase its environmental impact.

**Figure 5:**
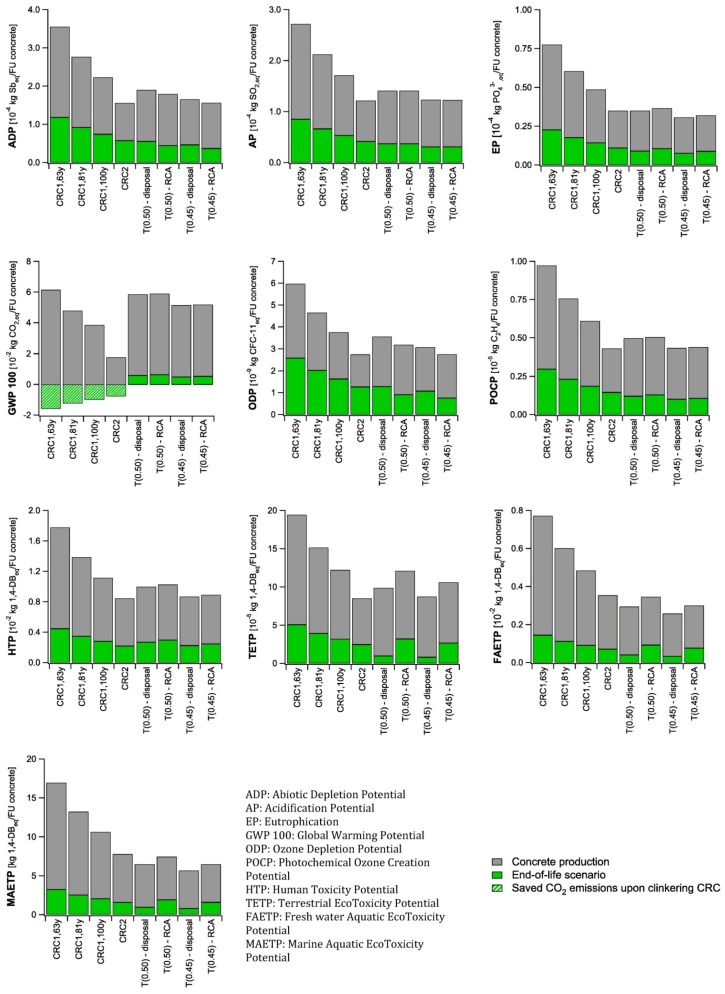
Cradle-to-cradle Life Cycle Impact Assessment of 1 FU concrete.

The question, thus, remains whether the whole CRC concept is indeed sustainable, when looking at its whole life cycle. The life cycle impact assessments of the different stages in the life cycle of concrete described in Section 4 were assembled for each concrete type, and subsequently multiplied by the corresponding functional unit calculated in Table 2. The obtained results are presented in Figure 5 and are used for the final assessment of the (potential) sustainability of CRC.

Looking at the results for traditional concrete, it is seen that the environmental impact in case of both recycling as aggregate or the disposal of the concrete is comparable regarding most impact categories (abiotic depletion, acidification, eutrophication, global warming, photo-oxidant formation and human toxicity). The impact of concrete disposal is higher when looking at the ozone layer depletion, while the impact on the ecotoxicity is higher for the recycling as concrete aggregate.

Both the low clinker content and the good strength performance of CRC2 compensate for the additional environmental costs related to its recycling as cement raw meal for most impact categories (abiotic depletion, acidification, eutrophication, global warming, ozone layer depletion, photo-oxidant formation, human toxicity and ecotoxicity). While in most cases, the impact of CRC2 is comparable to the traditional concrete mixtures, the benefits regarding the global warming potential are significant and a reduction of 66%–70% was calculated. The lower global warming potential for the CRC mixtures is the result of the lower CO_2_ emission of CRC raw material compared to traditional cement raw meals due to the presence of CO_2_ free lime in a CRC cement raw meal. The clinker content and strength performance of CRC1 are, however, not low or high enough, respectively, to obtain a lower environmental impact compared to the traditional concretes. Depending on the obtained service life, the environmental impact will be 5%–88% (100 years), 31%–134% (81 years), or 68%–200% (63 years) higher compared to the disposal of concrete and 1%–64% (100 years), 26%–104% (81 years), or 61%–161% (63 years) in comparison with recycling concrete as aggregate. Only regarding the global warming potential, the environmental impact might be 7%–35% lower if a service life between 81 and 100 years can be obtained. With a service life of 63 years, the global warming potential is 4%–20% higher compared to T(0.50) and T(0.45).

## 6. Conclusions

Completely Recyclable Concrete (CRC) is designed to be recycled within cement production, reducing the environmental impact of concrete and cement production. In order to prove the benefits for the environment of this CRC a life cycle assessment was conducted.

The environmental impact of the concrete production process was found to be strongly related to its binder content (mainly cement, but also fly ash by the economic allocation) and the required transport. Also the recycling opportunities of concrete are strongly affected by the required transport, which is in case of CRC expected to be higher, as transport to the cement plant is expected to be longer compared to the distance to a concrete recycling plant. The main environmental benefit of CRC recycling is related to its global warming potential. Compared to a traditional cement raw meal, CRC will contain a certain amount of CO_2_ free CaO. Thus making use of CRC instead of a traditional cement raw meal for the manufacturing of clinker will avoid the emission of a certain amount of CO_2_.

Looking at the complete life cycle of CRC and traditional concrete it was found that regarding the global warming potential a reduction of 66%–70% is possible when a high strength CRC with a low clinker content is designed. In case of a normal strength CRC with a higher clinker content, the reductions can be up to 7%–35% if a sufficient service life can be obtained. For most other impact categories (abiotic depletion, acidification, eutrophication, ozone layer depletion, photo-oxidant formation, human toxicity and ecotoxicity) only the performance of a high strength CRC with low clinker content could compensate for the additional transport required in the recycling process.
